# Safety and efficacy of oil palm phenolic supplementation in improving lipid profile among hyperlipidemic adults: a phase 2, randomized, double-blind, placebo-controlled clinical trial

**DOI:** 10.3389/fphar.2023.1190663

**Published:** 2023-07-07

**Authors:** Nur Balqis Muhammad Ismail Tadj, Nurul `Izzah Ibrahim, Tg Mohd Ikhwan Tg Abu Bakar Sidik, Mohamed S. Zulfarina, Qodriyah Haji Mohd Saad, Soon-Sen Leow, Syed Fairus, Isa Naina Mohamed

**Affiliations:** ^1^ Pharmacoepidemiology and Drug Safety Unit, Department of Pharmacology, Faculty of Medicine, Universiti Kebangsaan Malaysia Medical Centre, Kuala Lumpur, Malaysia; ^2^ Malaysian Palm Oil Board (MPOB), Kajang, Selangor, Malaysia

**Keywords:** oil palm phenolics, clinical trial, antihyperlipidemic, antioxidant, natural product

## Abstract

**Introduction:** Oil palm phenolic (OPP) is an antioxidant aqueous palm oil by-product and contains a high amount of phenolics. OPP has been proven to have many therapeutical benefits, and one of them is as an antihyperlipidemic agent. The previous phase 1 clinical trial proved OPP was safe to be orally consumed by healthy volunteers and yielded a good lipid profile. Thus, this phase 2 clinical trial was conducted to determine the effectiveness of OPP in improving the lipid profile among hyperlipidemic subjects.

**Methods:** A parallel, placebo-controlled, randomized, double-blinded clinical trial was conducted for 2 months on 50 hyperlipidemic subjects aged 20–50 years old. The subjects were randomly distributed to two treatment arms with 25 participants each: control/placebo (11 males and 14 females) and 250 mg of OPP (10 males and 15 females). The subjects were required to consume one capsule per day for 60 days. Fasting blood sampling for routine blood profile (hematology, liver function, renal function, and lipid) analysis and a medical examination were conducted at baseline, day 30, and day 60. *t*-test analysis was used to compare the difference between two test groups.

**Results:** The baseline lipid profile between control group (TC, 5.78 ± 0.52 mmol/L; LDL, 3.88 ± 0.51 mmol/L; HDL, 1.30 ± 0.25; TG, 1.30 ± 0.82), and 250 mg OPP (TC, 5.76 ± 0.54 mmol/L; LDL, 3.82 ± 0.59 mmol/L; HDL, 1.37 ± 0.34; TG, 1.25 ± 0.54) is insignificant. No serious adverse events (SAEs) were reported. No abnormality in fasting blood parameters in all groups was found. Compared to the control group among male participants, the 250 mg OPP group showed an improved serum triglyceride level. There were no statistically significant changes in all blood parameters from day 1 to day 60 with the exception of triglyceride level.

**Conclusion:** The absence of SAEs reported and no abnormal findings in biochemistry and hematology results suggested that the 250 mg OPP was safe to be taken by hyperlipidemic patients with a high probability of reducing triglyceride level in hyperlipidemic male patients The outcomes from this phase II trial suggest that by incorporating OPP supplements into the diet may be a promising strategy for individuals with hyperlipidemia to improve their lipid profiles and reduce cardiovascular risk. However, more research is needed to fully understand the mechanisms of action and establish the long-term efficacy and safety of OPP supplementation in larger scale.

**Limitation:** Small samples size hence lack of diversity (25 subjects per groups) and early sharing of treatment-response results.

**Clinical Trial Registration:** clinicaltrials.gov, identifier NCT04573218.

## 1 Introduction

Cardiovascular diseases (CVDs) remain a leading cause of death worldwide (WHO, 2021), including in Malaysia. The leading cause of deaths of Malaysians in 2021, apart from COVID-19, was ischemic heart diseases, which were 13.7% of the 157,251 medically certified deaths, followed by pneumonia (11.4%) and stroke (8.3%). Within a period of 5 years, the casualty by ischemic heart diseases increased by 90%, from 11,310 in 2016 to 21,543 in 2021 (Department of Statistic Malaysia, 2017; Department of Statistic Malaysia, 2022). Risk factors of CVDs are divided into two categories, modifiable and non-modifiable. The modifiable risk factors include hypertension, dyslipidemia, obesity, and diabetes, while non-modifiable risks are age, gender, and family history (Kucia and Hartley, 2022). The increasing cases of CVDs among Malaysians are correlated with the increasing prevalence of obesity, hypercholesterolemia, hypertension, and diabetes at the rate of 50.1%, 38.1%, 30.0%, and 18.3%, respectively, in 2019 (IPH, 2019).

Moreover, CVDs do not only endanger lives, but also economy and productivity. Malaysia is estimated to reach the status of an aged nation by 2030, with people over the age of 65 making up more than 14% of the population (WHO, 2022). Increases in elderly population might lead to increases number of CVDs, and the cost to treat CVDs and its related complications might increase as well. In 2017, CVDs alone caused the loss of RM 59.85 billion ($13.41 billion USD) productivity in Malaysia, and 67.4% of the loss occurred between the ages of 50 and 80 years (MOH, 2020). It is a known fact that CVDs mortality risk increases with age, and without early and effective interventions, the number of fatalities and economic burden will keep on snowballing. Consequently, treatments and care will become more challenging due to the lack of rehabilitation services in Malaysia (MOH, 2022).

Reactive oxygen species (ROS), defined as chemically unstable reactive free radicals containing oxygen, play a vital role in the progression of CVD as stated by Panth et al. (2016b). All the common risk factors of CVD (diabetes mellitus, smoking, aging and hypercholesterolemia) can further increase the possibility of ROS production. The same risk factors able to trigger several pathways such as apoptosis of endothelial cells (EC), expression of adhesion molecules, activation of metalloproteinases, induction of proliferation and migration of smooth muscle cells, lipid peroxidation, and change in vasomotor functions, collectively leading to CVDs (Vogiatzi et al., 2009; Panth et al., 2016a). For the majority of CVDs, the enzymatic sources of ROS include NAD(P)H oxidase, lipooxygenase, cyclooxygenase (COX), xanthine oxidase (XO), uncoupled nitric oxide synthases (NOS), cytochrome P450, and mitochondrial respiration (Coyle et al., 2006; Scherz-Shouval and Elazar, 2007; Paravicini and Touyz, 2008). Overexpression of ROS can lead to various negative effects as stated before but at the physiological level, ROS is very important to promotes cellular activities, controls the hormone level, maintains chemical balance, strengthens synaptic plasticity, fight against invading pathogens and induce an immune response against the pathogenic influence (Zuo et al., 2015). Normally, ROS can be neutralized by intracellular antioxidant enzymes such as glutathione peroxidase (GPx), superoxide dismutase (SOD), and catalase and consumption of other non-enzyme antioxidants like *β*-carotene, ascorbic acid, and tocopherols as a supplement (Wattanapitayakul and Bauer, 2001).

Hypercholesterolemia, or also termed as hyperlipidemia, refers to the imbalance of lipids such as cholesterol, low-density lipoprotein (LDL), triglycerides, and high-density lipoprotein (HDL) (Pappan and Rehman, 2022). Hyperlipidemia is one the CVDs risk factors with a high prevalence among Malaysians involving 64.0% and 56.7% of Malaysian adults who experienced elevated total cholesterol (TC) and LDL, respectively (Mohamed-Yassin et al., 2021). Although lipid-lowering medication (LLM) especially statin is recommended for secondary prevention (for patients with existing CVDs) and primary prevention (for those with high risks to develop CVDs) (MOH, 2017), they are underutilized. Only 25.8% of patients with existing CVDs and 10.0% of patients who had a high CVDs risk were on LLM for secondary prevention and primary prevention, respectively. The low utilization of LLM is especially prevalent among the less-educated, low-income earners and rural residents (Baharudin et al., 2022). Natural products are one the traditional and complementary medicines (TCMs) that are frequently used to treat and prevent illnesses. The elderly, low-income earners, and villagers usually used natural products after being diagnosed with hypercholesterolemia (Abdullah et al., 2018), while highly educated people with higher income and live in the urban areas used natural products to maintain their general health (Lim et al., 2005; Hasan et al., 2009). The popularity of natural products especially among elderly might be due to the perception that the natural products are cheaper, safer, and more effective compared to the modern medicines (Welz et al., 2018).

There are various treatments option in treating CVD available nowadays, either using modern medicine and complementary medicine or practicing healthy lifestyle. While modern medicines are the methods that are commonly used, there are a few challenges faced by the consumers, especially those in developing countries such as Malaysia. A few studies reported that declining number of patients consuming modern CVD drugs might due to concerns about the cost of lifelong treatment (Ramiro et al., 2000; Beshir et al., 2016) and side effects (Lee et al., 2014; Shima et al., 2014; Risso-Gill et al., 2015). They also were concerned that they might become dependent on medication, or suspected that prolonged use of medication might cause side effects hence many will resort to complementary medicine. Dietary supplements (fish oil, coenzyme Q10, garlic, etc.) are among the most commonly used treatment modalities in patients with CVD. Fish oil supplements are accepted as a part of the treatment regimen for elevated serum triglycerides and the maintenance of vascular wall health. However, the efficacy of vitamin E has been questionable (Qidwai et al., 2013). There are a few CVD treatments require long-term monitoring and some places (especially urban areas) are difficult to reach for the appointment, hence many will just stop with their treatment (Hanafi et al., 2015). Apart from consuming medicines, patients are advised to practice healthy lifestyle to improve their heart conditions such as exercise regularly, maintaining a healthy weight, following a balanced diet, limiting alcohol consumption, quitting smoking, and managing stress.

Oil palm phenolic (OPP) was developed by the Malaysian Palm Oil Board (MPOB) as one of the efforts to maximize the usage of palm oil in Malaysia. The manufacturing process was patented by Sambanthamurthi et al. (2008). Since then, various research efforts were performed to build the pharmacological profiles of OPP. High-performance liquid chromatography (HPLC) and nuclear magnetic resonance (NMR) analyses show that OPP is rich with phenolic contents, majorly contributed by caffeoylshikimic acid (CFA), followed by p-hydroxybenzoic acid (PHBA) and protocatechuic acid (PCA) (Sambanthamurthi et al., 2011). Moreover, OPP is not only a powerful antioxidant (Balasundram et al., 2005; Syarifah-Noratiqah et al., 2019), but it is also effective as an antitumor (Sekaran et al., 2010), antiatherogenic (Idris et al., 2014), antidiabetic (Bolsinger et al., 2014), antiamyloidogenic (Monplaisir, 2016), and antihyperlipidemic agent (Fairus et al., 2018). The 90-day *in vivo* animal toxicity study showed that OPP had no observable adverse effects in human in an equivalent dose of up to 2,000 mg/kg body weight per day. No significant effects were also noted on body weight, food consumption, hematology, clinical chemistry, organ weights, and histopathological examination (Lynch et al., 2017).

There are several mechanisms of OPP that may help in improving cardiovascular health such as biosynthesis of cholesterol, antioxidant and anti-inflammatory. Downregulation of cholesterol biosynthesis genes (Hmgcs1, Lss, Sc4mol, Fdps, Nsdhl) in BALB/c mice might exert a hypocholesterolemia effect (Leow et al., 2011). The OPP supplementation also revealed negative fold change in the expression of HMGCR gene, a statin-targeted gene responsible for lowering cholesterol level. Another study of OPPP with atherogenic-diet fed rabbits, the results show that there was a significant reduction of fatty streaks and plaques (Idris et al., 2014). The upregulation of antioxidant gene expression such as Mgst1, Gpx1 (Leow et al., 2013), Gstm2, Gstm5 and Gstm6 are highly involve with scavenging the ROS. The upregulated genes imply that OPP confers a great ability to fight against oxidative stress in the heart, which is susceptible to prooxidant exposure (Leow et al., 2011). The same author also demonstrated that the OPP has modulated Helper T-cell (Th) of the immune system towards the cytokines that possess anti-inflammatory actions. This modulation in turn contribute to the reduction of atherosclerosis development. Atherogenic-diet fed mice that was given OPP had significantly reduced pro-inflammatory IL-12 cytokine while significantly increased anti-inflammatory IL-13 cytokine (Leow et al., 2013). The changes in cytokine levels may promote the anti-inflammatory response, thus preventing atherosclerosis development. IL-12, an innate immunity cytokine, has been implicated in atherosclerosis and other inflammatory diseases (Kleemann et al., 2008; Abbas et al., 2014).Clinical trials have been conducted to determine if OPP has the potential to be developed and marketed as antihypercholesterolemic health supplements. Two clinical trial phases were performed to evaluate the safety of OPP in Malaysia. Both trials were conducted among healthy participants for 60 days. The first clinical trial was a single-blinded trial run by Fairus et al. (2018) where the participants were required to drink 9,000 mg/kg OPP daily in the form of juice. Their results showed no record of serious adverse events (SAEs). We conducted the second clinical trial phase 1 with several adjustments: a double-blinded study, using multiple doses (low, middle, and high doses), reducing the maximum dosage from 9,000 mg to 1,500 mg, and the OPP extract was encapsulated for easy consumption and to increase compliance rate. The results from our phase 1 safety study also showed no SAEs, absence of abnormality findings in biochemistry and hematology results, and 250 mg OPP was determined as the optimum dosage (Muhammad Ismail Tadj et al., 2022). As the safety of OPP consumption among healthy human subjects was clinically proven, we then proceeded to the phase 2 to determine the safety and efficiency of OPP in improving the lipid profile in the hyperlipidemic population. The finding from our phase 2 clinical trial is important in order to proceed with further trials. Moreover, if OPP is proven to be effective as anti-hyperlipidemic supplements, this can make situation convenient for the patients to add OPP as part of their CVD treatments.

## 2 Materials and methodology

### 2.1 Study drug composition

The green-colored 250 mg OPP extract used in this trial contained active ingredients of hydroxytyrosol (C_8_H_10_O_3_), p-hydroxybenzoic acid (C_7_H_6_O_3_), protocatechuic acid (C_7_H_6_O_4_), and caffeoylshikimic acid (C_16_H_16_O_8_) as patented by Sambanthamurthi et al. (2011). The dose was chosen based on the previous clinical trial (Muhammad Ismail Tadj et al., 2022). The placebo/control used in this trial was dextrose sugar. Both OPP extract and control were encapsulated in white-opaque capsule for easy consumption. Both OPP and control capsules were identically packaged by the appointed contract research organization (CRO), and they were only dispensed by blinded trial team members.

### 2.2 Study design and protocol

This clinical trial was a mono-centric, parallel, placebo-controlled, randomized, double-blind phase 2 study conducted to study the effectiveness of OPP in improving lipid profile among hyperlipidemic subjects. This trial was conducted at the clinical trial ward (CTW), Hospital Canselor Tuanku Muhriz (HCTM), Kuala Lumpur. The trial was designed and performed according to Good Clinical Practice (GCP), Declaration of Helsinki, and Malaysian GCP Guidelines. The approval to conduct this trial was given by the Research Ethics Committee of Universiti Kebangsaan Malaysia (RECUKM) (UKM PPI/111/8/JEP-2019–100). The study protocol was also registered at clinicaltrials.gov under the registration number NCT04257929.

**FIGURE 1:**
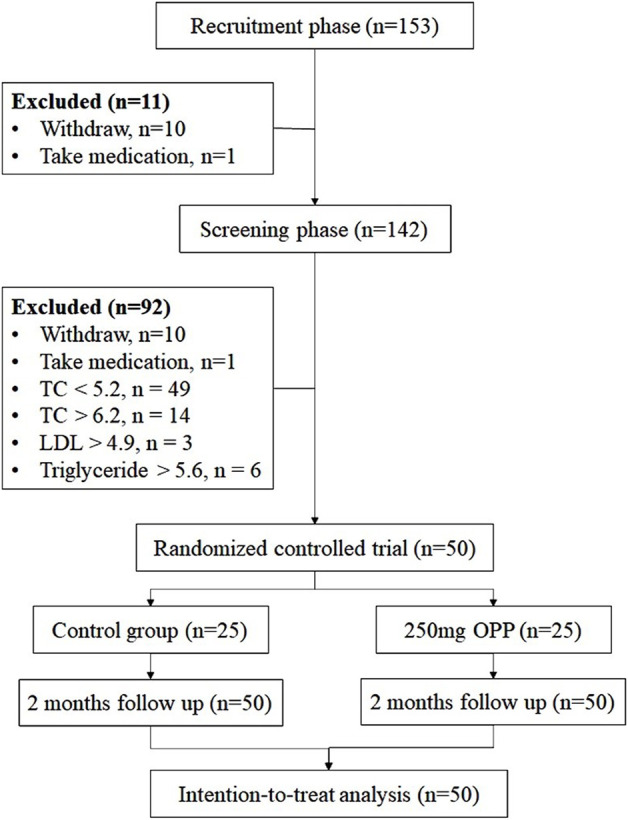
CONSORT diagram showing trial and participant flow through the trial.

This OPP phase 2 trial consisted of three stages, namely, recruitment, screening, and 2 months intervention, as illustrated in Figure 1. The recruitment was conducted for several weeks starting in October 2022 by using multiple approaches (i.e., email, poster, flyers, and social networks). Interested participants were screened based on inclusion/exclusion criteria (as described in Section 2.3 Inclusion and Exclusion Criteria). Before proceeding into the trial, all the volunteers were briefed about the study protocol and procedures. Only after they fully understood and submitted their informed consent forms, their blood was taken and medical examinations (medical history, allergies, drug/supplements intake) were performed by the appointed physicians. Qualified volunteers were randomly assigned into two groups with 25 volunteers in each group. The groups were placebo/control group and 250 mg OPP group. The volunteers were required to consume one capsule per day for 2 months. Assignments into each study group remained concealed until the study was completed.

The study visits are tabulated in Table 1. This trial had two sessions, intervention and non-intervention. The intervention session was on baseline/day 1, day 30, and day 60, while non-intervention was during the daily and weekly visits. The intervention session involved blood sampling and medical examination, hence the attendance of the volunteers was compulsory. The flow during the intervention day started with registration, vital signs (pulse and blood pressure), height and weight measurements, blood sampling, consuming the capsule in front of the study staff, restocking capsule supply (except for day 60), and finally a medical examination. All volunteers were reminded to fast overnight prior to the day before the intervention day for collecting their fasting blood sampling. Vital signs as well as height and weight measurements were taken by the CTW nurses. Then, the volunteers were directed into the blood sampling room. 20 mL of blood was withdrawn by a phlebotomist using a butterfly syringe and transferred into ethylenediamine tetra acetic acid (EDTA) and plain tube for hematology and biochemistry analyses, respectively. The blood was then analyzed by a certified independent laboratory on the same day. With the exception on day 60, all volunteers were required to consume the capsule in front of the study staff and to restock their weekly supply before meeting with the physicians. A thorough medical examination by the physicians on duty was conducted. A series of questions were asked, and a physical examination was performed. All responses were recorded in the case report form (CRF).

**TABLE 1:** Study visit phase 2.

Action visit	V1^#^	V2	V3	V4	V5	V6	V7	V8	V9^#^	V10	V11	V12	V13^#^
Day	1	2	3	4	5	9	16	23	30	37	44	51	60
Briefing	x												
Informed consent	x												
Subject Diary^a^	x												
Case Report Form (CRF)^b^	x												
Vital sign (BP, pulse)	x	x	x	x	x	x	x	x	x	x	x	x	x
Height measurement	x	x	x	x	x	x	x	x	x	x	x	x	x
Weight measurement	x	x	x	x	x	x	x	x	x	x	x	x	x
Blood sampling	x								x				x
Capsule consumed in front of trial staffs	x	x	x	x	x	x	x	x	x	x	x	x	
Restock stock supply^c^					x	x	x	x	x	x	x	x	
Medical examination and doctor consultation	x								x				x
Adverse event assessment^d^	x	x	x	x	x	x	x	x	x	x	x	x	x

Abbreviation: BP, (Blood pressure).

^**#**^Intervention days (day 1, day 30, day 60): Volunteers were reminded a day prior to fast for 8 h before appointment time for blood collection. Medical examination and doctor consultation were mandatory. Data was recorded in CRF, distributed on day 1.

^a^ Subject diary was given to be kept by the volunteers to record the time the capsule was consumed, reason(s) if the capsule was not taken within the 60 days, intake of another drug/supplement and the reason(s).

^b^ Case Report Form (CRF) is an official document to record demographic information, medical history, drugs/supplements intake, adverse events occurred during the trials (if any), and other important information. CRF was first given to the volunteers to ensure demographic information was correctly written. The volunteers returned the CRF during their first medical examination to the appointed physicians, and the CRF was stored in the CTW throughout entire trial duration.

^c^ The volunteers were not required to come on weekend, hence supply was given on Friday (first week). Then, starting from the second week, the volunteers were only required to come once a week to get a 1-week stock supply.

^d^ Any adverse events that occurred throughout the entire trial period were investigated and recorded into CRF.

**FIGURE 2:**
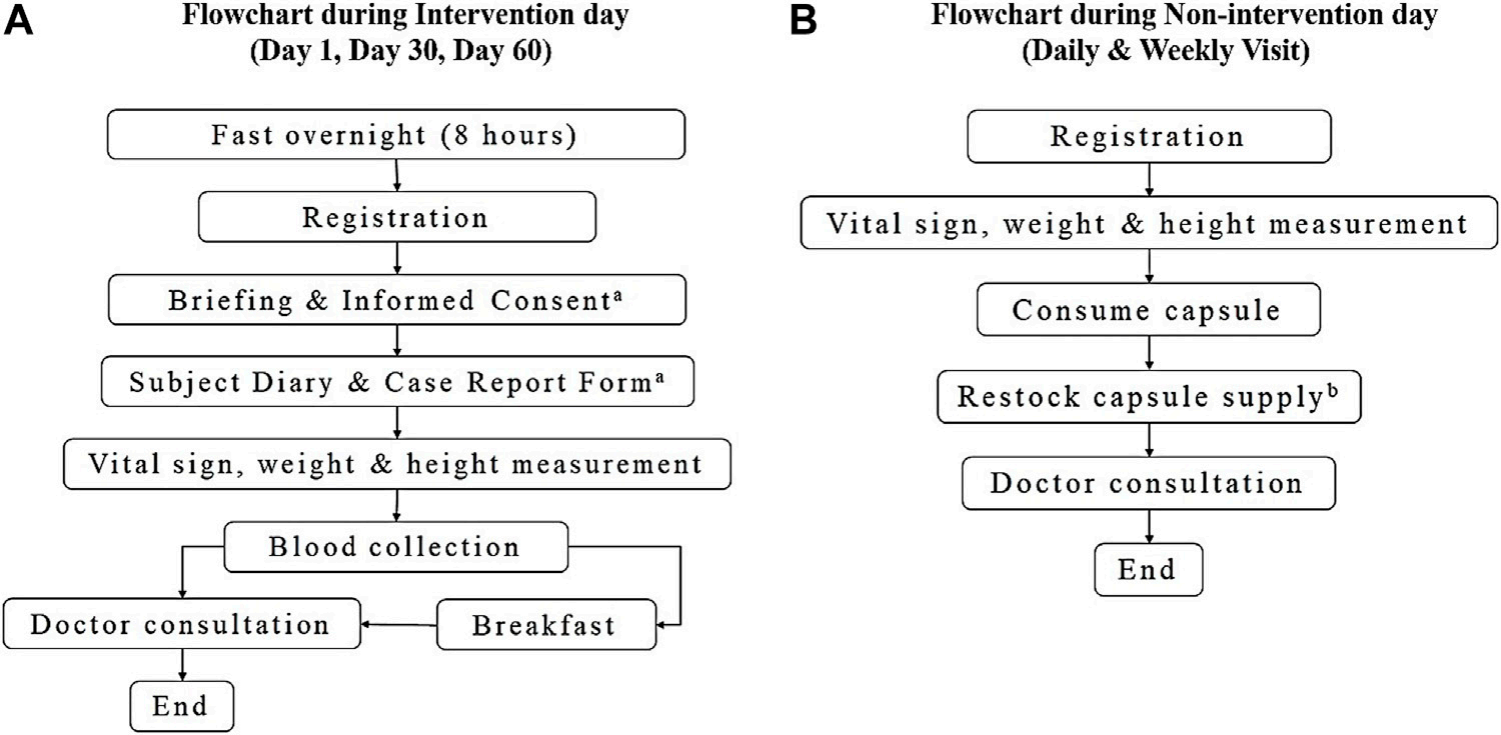
Flowchart during intervention and non-intervention session.

The flow for non-intervention day was similar to the intervention day but excluding the mandatory physical examination and blood sampling. Daily attendance during the first week was mandatory. This was important for monitoring the presence of any adverse effects after the volunteers consumed the capsule. As for the weekly visit, the volunteers were asked to be present on the appointed day to restock their weekly capsule supply. If they were unable to come, another appointment day would be arranged on the same week. Doctor consultation was not compulsory. However, medical physicians were available upon request during each visit. The volunteers were required to write down the timing when they took their daily capsule on the subject diary given on day 1 to ensure drug compliance. The flowchart for both intervention and non-intervention days is illustrated as in Figure 2.

### 2.3 Inclusion and exclusion criteria

#### 2.3.1 Inclusion criteria

1. Male and female volunteers aged 20–50 years at time of consent
2. Has mild fasting blood lipid profile:
  i. Total cholesterol 5.2 − 6.2 mmol/L
  ii. LDL cholesterol 3.4 − 4.9 mmol/L
  iii. Triglycerides 1.7 − 5.6 mmol/L

#### 2.3.2 Exclusion criteria

1. Smokers and vapers
2. Habitual alcohol consumption (once a month was acceptable)
3. Actively consuming antioxidant supplements or lipid-lowering medication (e.g., statin)
4. Pregnant or breastfeeding mother
5. Had a medical history of cardiovascular disease, diabetes, or dyslipidemia

### 2.4 Sample size and randomization

Like in the previous phase 1 trial, Sakpal (2010) was referred to in the sample size calculation. A minimum number of 19 volunteers per arm were needed, assuming two-sided testing at a significant level of 5% and 80% power. Considering the possibility of dropout, we recruited a total of 50 volunteers with 25 volunteers per arm.

Stratified randomization was performed at a ratio of one to one into OPP vs. control by using statistical software, Stata 14.0. Participant randomization was stratified by age, gender, and total cholesterol. The distribution of age and total cholesterol was approximately normal. Therefore, the volunteers were divided into two groups based on the mean value. In total, there were eight subgroups according to these three characteristics. The allocation sequence for all subgroups was generated by using a computer-generated list (seed number 123456).

### 2.5 General measurement

The volunteers were asked to rest before their vital signs (pulse and blood pressure) were measured by using the Advanced^®^ VSM-300 Vital Signs Monitor. Then, their height and weight were measured by using Adam Equipment MDW-250 L Digital Physician Scale. Both measurements were conducted by the CTW nurses.

### 2.6 Fasting blood parameters

The fasting blood samples were sent to an independent laboratory diagnosis center (PATHLAB) to be processed and analyzed for hematology and biochemistry profiling (renal blood profile, liver blood profile, and lipid profile).

The blood samples that were collected into EDTA tubes were analyzed for hematology parameters using CELL-DYN Ruby Hematology Analyzer. The measured parameters were erythrocyte sedimentation rate (ESR), red blood cells (RBCs), hemoglobin (Hb), packed cell volume (PCV), mean corpuscular volume (MCV), mean corpuscular hemoglobin (MCH), mean corpuscular hemoglobin concentration (MCHC), platelet count, white blood cells (WBCs), neutrophils, lymphocytes, monocytes, eosinophils, and basophils.

For biochemistry profiling, the blood samples were collected in plain tubes without any anticoagulation. The serum was then analyzed using Cobas^®^ 6,000 Chemistry Analyzer. Measured renal parameters were serum urea, creatinine, calcium, phosphate, uric acid, sodium, potassium, and chloride. The liver parameters measured were total protein (TP), albumin, globulin, bilirubin, alkaline phosphatase (ALP), aspartate transaminase (AST), alanine transaminase (ALT), and gamma-glutamyl transferase (GGT). Finally, the lipid parameters measured were total cholesterol (TC), high-density lipoprotein (HDL), low-density lipoprotein (LDL), and triglycerides (TG).

### 2.7 Adverse event assessments and reporting

The physical examination and medical history conducted on day 1 was performed to help with interpretation of adverse events (AEs) or serious adverse events (SAEs) reported by the volunteers throughout the entire trial. The volunteers were recommended to report any discomforts they felt at any time, whether by face-to-face visits or through phone calls. The specifics of each event such as duration, severity, and onset were investigated by the physicians and were recorded in CRF regardless of whether the events were related to OPP consumption. The in-charge physicians then decided if the study intervention should be temporarily or permanently stopped. All AEs would be actively followed up until the condition was stabilized, especially if the AEs caused discontinuation of study intervention. In the case of SAEs reported, the sponsor and the sponsor representative were notified within 24 h. Any Suspected, Unexpected Serious Adverse Reactions (SUSARs) would be informed to the appropriate regulatory authorities following local and global guidelines and requirements.

### 2.8 Statistical analysis

Statistical Package for the Social Sciences (SPSS), version 22.0 was used to conduct the statistical analysis. Intention to treat analysis (ITT) was used to analyze the primary outcomes. Missing variables were assigned using simple imputation. Continuous normally distributed data were presented as the mean and standard deviation (SD), while not normally distributed data were presented as the median and interquartile range (IQR).

A one-way ANOVA test was used to examine the difference in safety profile values between control and 250 mg OPP groups (between-subjects) at baseline, 30 days, 60 days, and the average (combination of all three values). Repeated measure ANOVA was used to compare the changes between time periods within each group (within-subjects). A *p*-value of less than 0.05 was considered statistically significant.

## 3 Results

### 3.1 Baseline demographic

Table 2 shows the baseline characteristics of subjects who completed 2 months of treatment, stratified by age, gender, and lipid profiles. The subjects in both groups were considered obese following the Asian BMI cut-off points, where 27.5–32.4 kg/m^2^ was considered obese (MIMS, 2022). The means for TC, LDL, HDL, and TG in the control group were 5.78, 3.88, 1.30, and 1.30 mmol/L, respectively. Meanwhile, in the 250 mg OPP group, the means for TC, LDL, HDL, and TG were 5.76, 3.82 , 1.37, and 1.25 mmol/L, respectively. Fisher’s exact tests showed no significant difference between the two groups.

**TABLE 2:** Baseline characteristics of 50 hyperlipidemic volunteers in phase 2.

Parameters	Control (n = 25)	250 mg OPP (n = 25)	*p*
Gender/Sex			
Male (n)	11	10	0.774
Female (n)	14	15	
Age (years)	33.8 ± 7.4	33.5 ± 6.3	0.861
BMI (kg.m^−2^)	27.29 ± 5.12	28.31 ± 7.18	0.566
Systolic (mmHg)	122.4 ± 11.4	124.6 ± 13.3	0.532
Diastolic (mmHg)	79.6 ± 10.9	79.1 ± 8.7	0.864
Total cholesterol (mmol/L)	5.78 ± 0.52	5.76 ± 0.54	0.895
LDL (mmol/L)	3.88 ± 0.51	3.82 ± 0.59	0.664
HDL (mmol/L)	1.30 ± 0.25	1.37 ± 0.34	0.409
Triglycerides (mmol/L)	1.30 ± 0.82	1.25 ± 0.54	0.808

Abbreviations: BMI, body mass index; LDL, low-density lipoprotein; HDL, high-density lipoprotein. Data are tabulated as mean ± standard deviation or n.

### 3.2 Safety assessment

The results for the safety assessment between control and OPP groups for all the 50 subjects are tabulated in Table 3. None of the subjects had SAEs that required the target supplement to be temporarily or permanently stopped. Minor adverse effects reported in the control group were increased appetite (n = 5), light-headedness (*n* = 2), and sleepiness (*n* = 1), while in the 250 mg OPP group, four volunteers reported increased appetite and two reported 2 days of being light-headed. There was no statistically significant difference in the reporting of undesirable side effects in the 250 mg OPP group as opposed to the control group.

**TABLE 3:** Safety assessment reported by volunteers within 2 months of the trial.

Symptoms	All (n = 50)	Control (n = 25)	250 mg OPP (n = 25)	*p*
None reported	7	5	2	0.417
Bloated	1	0	1	1.000
Easy to defecate	13	6	7	0.747
Get hungry easily	9	5	4	1.000
Lightheaded	4	2	2	1.000
Lose weight easily	5	3	2	1.000
More energetic	7	3	4	1.000
Reduce menstrual pain	1	0	1	1.000
Short menstrual duration	2	0	2	0.490
Sleepiness	1	1	0	1.000

Data are tabulated as frequency, n (%).

### 3.3 Body weight

The body weight results of the hyperlipidemic subjects on day 1, day 30, and day 60 during phase 2 are tabulated in Table 4. Both control and 250 mg OPP groups showed consistent body weight throughout the entire trial. The results of repeated measure ANOVA for the control group (*p* = 0.596) and the 250 mg OPP group (*p* = 0.103) were insignificant. The difference between the groups was also insignificant (*p* = 0.328).

**TABLE 4:** Mean body weight (kg) of hyperlipidemic volunteers during phase 2.

	Control (n = 25)	250 mg OPP (n = 25)	*p* ^1^
Day 1	69.47 ± 12.99	73.68 ± 18.78	0.361
Day 30	69.49 ± 13.03	74.09 ± 19.29	0.328
Day 60	69.26 ± 12.78	74.09 ± 19.08	0.299
*p* ^2^	0.596	0.103	

^1^Independent *t*-test between group, *p* < 0.05 as statistically significant.

^2^Repeated measure ANOVA, within the group, *p* < 0.05 as statistically significant.

Data are tabulated as mean ± standard deviation.

### 3.4 Hematology parameters

The results for fasting hematology parameters are displayed in Table 5. As shown in the table, MCH significantly decreased (*p* = 0.026) in the control group, while in the 250 mg OPP group, RBC (*p* = 0.008) and PCV (*p* = 0.049) significantly increased from baseline to day 60. Although the three parameters were statistically different within the groups, when a *t*-test analysis between the group was performed, the results showed no statistically significant difference. The same results were found for the rest of parameters, with an exception of the WBC parameter. On day 30, WBC for 250 mg OPP group was significantly lower than the control group (*p* = 0.013), although the reduction in WBC was still within the normal range. All of the measured parameters were within normal clinical ranges.

**TABLE 5:** Hematology parameter values of hyperlipidemic volunteers at baseline, day 30, and day 60 during phase 2 (mean ± SD).

	Control (n = 25)	250 mg OPP (n = 25)
ESR (mm/hr)		
Day 1	15.96 ± 12.21	14.52 ± 9.92
Day 30	12.96 ± 12.40	12.52 ± 9.19
Day 60	14.20 ± 11.57	14.80 ± 11.46
RBCs (10^12^/L)		
Day 1	4.92 ± 0.58	4.80 ± 0.45
Day 30	4.94 ± 0.54	4.89 ± 0.51^a^
Day 60	4.91 ± 0.52	4.92 ± 0.45^a^
Hb (g/dL)		
Day 1	13.78 ± 1.55	13.64 ± 1.34
Day 30	13.76 ± 1.56	13.82 ± 1.52
Day 60	13.68 ± 1.54	13.84 ± 1.32
PCV (%)		
Day 1	40.64 ± 4.17	40.80 ± 3.62
Day 30	40.76 ± 4.14	41.60 ± 4.08^a^
Day 60	40.68 ± 4.27	41.64 ± 3.62^a^
MCV (fL)		
Day 1	83.28 ± 5.95	85.04 ± 4.84
Day 30	83.00 ± 5.93	85.04 ± 4.76
Day 60	83.36 ± 6.36	85.00 ± 4.86
MCH (pg)		
Day 1	28.40 ± 2.45	28.40 ± 1.94
Day 30	28.04 ± 2.39	28.28 ± 1.86
Day 60	27.96 ± 2.37^a^	28.24 ± 1.92
MCHC (10^9^/L)		
Day 1	33.92 ± 1.38	33.28 ± 0.68
Day 30	33.72 ± 1.06	33.32 ± 0.90
Day 60	33.64 ± 1.19	33.32 ± 0.75
Platelet (10^9^/L)		
Day 1	308.12 ± 96.03	285.28 ± 53.29
Day 30	313.36 ± 94.05	295.92 ± 51.94
Day 60	306.72 ± 82.53	292.84 ± 56.86
WBCs (10^9^/L)		
Day 1	7.46 ± 2.15	6.50 ± 1.44
Day 30	7.37 ± 1.38	6.38 ± 1.33*
Day 60	7.29 ± 1.36	6.50 ± 1.63
Neutrophil (%)		
Day 1	56.15 ± 8.87	52.87 ± 8.18
Day 30	56.00 ± 5.86	52.49 ± 7.94
Day 60	57.20 ± 9.36	52.07 ± 8.61
Lymphocytes (%)		
Day 1	34.18 ± 7.84	37.00 ± 7.42
Day 30	34.28 ± 5.22	37.17 ± 7.01
Day 60	33.58 ± 8.42	37.20 ± 7.30
Monocytes (%)		
Day 1	5.94 ± 1.57	6.51 ± 1.48
Day 30	6.32 ± 1.56	6.54 ± 1.55
Day 60	6.13 ± 1.45	6.78 ± 1.42
Eosinophils (%)		
Day 1	3.17 ± 2.93	3.01 ± 1.94
Day 30	2.84 ± 1.64	3.17 ± 2.03
Day 60	2.54 ± 1.39	3.30 ± 2.48
Basophil (%)		
Day 1	0.56 ± 0.24	0.61 ± 0.20
Day 30	0.56 ± 0.16	0.64 ± 0.31
Day 60	0.55 ± 0.20	0.64 ± 0.27

^a^Significantly different compared to day 1 within the group (*p* < 0.05).

^b^Significantly different compared to day 1 within the group (*p* < 0.001).

*Significantly different compared to control group (*p* < 0.05).

### 3.5 Renal function test (RFT)

The results of fasting serum RFT parameters are displayed in Table 6. The majority of the parameters in both control and 250 mg OPP groups showed the same increase and decrease patterns within the groups. Hence, none of the parameters was statistically significant different when compared between each other using *t*-test. Creatinine significantly increased in the control group (*p* < 0.001) and the 250 mg OPP group (*p* = 0.003) from day 1 to day 30, and then the level insignificantly decreased until day 60. Calcium in both groups increased from day 1 to day 60, although only in the 250 mg OPP group the rise was statistically significant (*p* = 0.020). Uric acid in the control group significantly decreased (*p* < 0.001) in the last 30 days of trial, while the 250 mg OPP group also showed a decrease but the changes were insignificant. Potassium in both groups was low in the first 30 days albeit not statistically significant. But, the value significantly rose in both control group (*p* = 0.037) and 250 mg OPP group (*p* = 0.026) from day 30 to day 60. Chloride significantly dropped in both control group (*p* = 0.038) and 250 mg OPP group (*p* < 0.001) in the first 30 days. Then, the value became stagnant until the trial ended. Despite all the statistically significant changes shown by the renal parameters within the 2 months, none of the value fell outside the normal clinical ranges.

**TABLE 6:** Serum renal function test of hyperlipidemic volunteers at baseline, day 30, and day 60 during Phase 2 (mean ± SD).

	Control	250 mg OPP
Urea (mmol/L)		
Day 1	4.03 ± 0.99	4.27 ± 1.15
Day 30	4.30 ± 0.80	4.46 ± 1.11
Day 60	4.01 ± 0.95	4.34 ± 1.10
Creatinine (mmol/L)		
Day 1	69.44 ± 16.74	69.88 ± 16.35
Day 30	73.40 ± 16.67^b^	73.68 ± 17.32^a^
Day 60	71.76 ± 16.63^a^	73.04 ± 15.38^a^
Calcium (mmol/L)		
Day 1	2.24 ± 0.08	2.23 ± 0.08
Day 30	2.26 ± 0.07	2.31 ± 0.11^a^
Day 60	2.28 ± 0.06	2.31 ± 0.08^a^
Phosphate (mmol/L)		
Day 1	1.14 ± 0.12	1.14 ± 0.18
Day 30	1.14 ± 0.14	1.18 ± 0.18
Day 60	1.15 ± 0.14	1.17 ± 0.16
Uric acid (mmol/L)		
Day 1	0.35 ± 0.08	0.36 ± 0.10
Day 30	0.36 ± 0.09	0.36 ± 0.10
Day 60	0.33 ± 0.09^c^	0.35 ± 0.10
Sodium (mmol/L)		
Day 1	137.92 ± 1.12	138.84 ± 1.37
Day 30	137.28 ± 1.31	137.76 ± 1.45^a^
Day 60	137.56 ± 1.76	137.52 ± 1.53
Potassium (mmol/L)		
Day 1	4.38 ± 0.43	4.33 ± 0.39
Day 30	4.36 ± 0.34	4.32 ± 0.38
Day 60	4.60 ± 0.42^c^	4.50 ± 0.40^c^
Chloride (mmol/L)		
Day 1	105.24 ± 1.54	105.88 ± 1.86
Day 30	103.92 ± 2.08^a^	103.48 ± 2.00^b^
Day 60	104.00 ± 1.35	103.76 ± 1.54

^a^Significantly different compared to day 1 within the group (*p* < 0.05).

^b^Significantly different compared to day 1 within the group (*p* < 0.001).

^c^Significantly different compared to day 30 within the group (*p* < 0.05).

### 3.6 Liver function test (LFT)

The results of fasting serum LFT parameters are displayed in Table 7. There was no significant difference in both control and 250 mg OPP groups. The ALT level in both groups increased from day 1 to day 30. However, the changes in the control group (*p* = 0.037) were significant, while the changes in the 250 mg OPP were insignificant. Next, the GGT level for both groups increased in the first 30 days. However, only the 250 mg OPP group showed a significant change (*p* = 0.01). Then, the GGT level dropped in both groups until day 60, with significant reduction in the control group (*p* = 0.037). All the measured values were still within the normal clinical ranges.

**TABLE 7:** Serum liver function test of hyperlipidemic volunteers at baseline, day 30, and day 60 during phase 2 (mean ± SD).

		Control	250 mg OPP
Total protein			
Day 1		72.24 ± 3.74	70.92 ± 3.03
Day 30		72.24 ± 3.60	72.08 ± 4.55
Day 60		71.96 ± 4.10	72.36 ± 4.23
Albumin			
Day 1		44.88 ± 2.01	44.76 ± 1.94
Day 30		44.84 ± 1.68	45.04 ± 2.75
Day 60		44.84 ± 1.80	45.80 ± 2.38
Globulin			
Day 1		27.36 ± 3.08	26.16 ± 2.62
Day 30		27.40 ± 3.45	27.04 ± 3.23
Day 60		27.12 ± 3.35	26.56 ± 3.06
ALP (IU/L)			
Day 1		67.48 ± 15.38	75.24 ± 22.65
Day 30		65.16 ± 15.70	75.48 ± 19.87
Day 60		65.88 ± 15.35	76.72 ± 20.28
AST (IU/L)			
Day 1		20.80 ± 7.54	22.32 ± 5.84
Day 30		22.88 ± 11.65	23.12 ± 6.30
Day 60		21.20 ± 8.76	23.16 ± 4.37
ALT (IU/L)			
Day 1		20.92 ± 15.49	21.56 ± 11.15
Day 30		25.40 ± 18.83^a^	25.88 ± 17.21
Day 60		24.16 ± 18.90^a^	25.16 ± 14.32
GGT (IU/L)			
Day 1		22.40 ± 11.73	23.84 ± 16.01
Day 30		25.16 ± 10.55	28.72 ± 22.14^a^
Day 60		22.76 ± 10.23^c^	26.12 ± 17.48

Abbreviation: ALP, alkaline phosphatase; AST, aspartate transaminase; ALT, alanine transaminase; GGT, gamma-glutamyl transferase.

^a^Significantly different compared to day 1 within the group (*p* < 0.05).

^b^Significantly different compared to day 1 within the group (*p* < 0.001).

^c^Significantly different compared to day 30 within the group (*p* < 0.05).

### 3.7 Lipid profile

**FIGURE 3:**
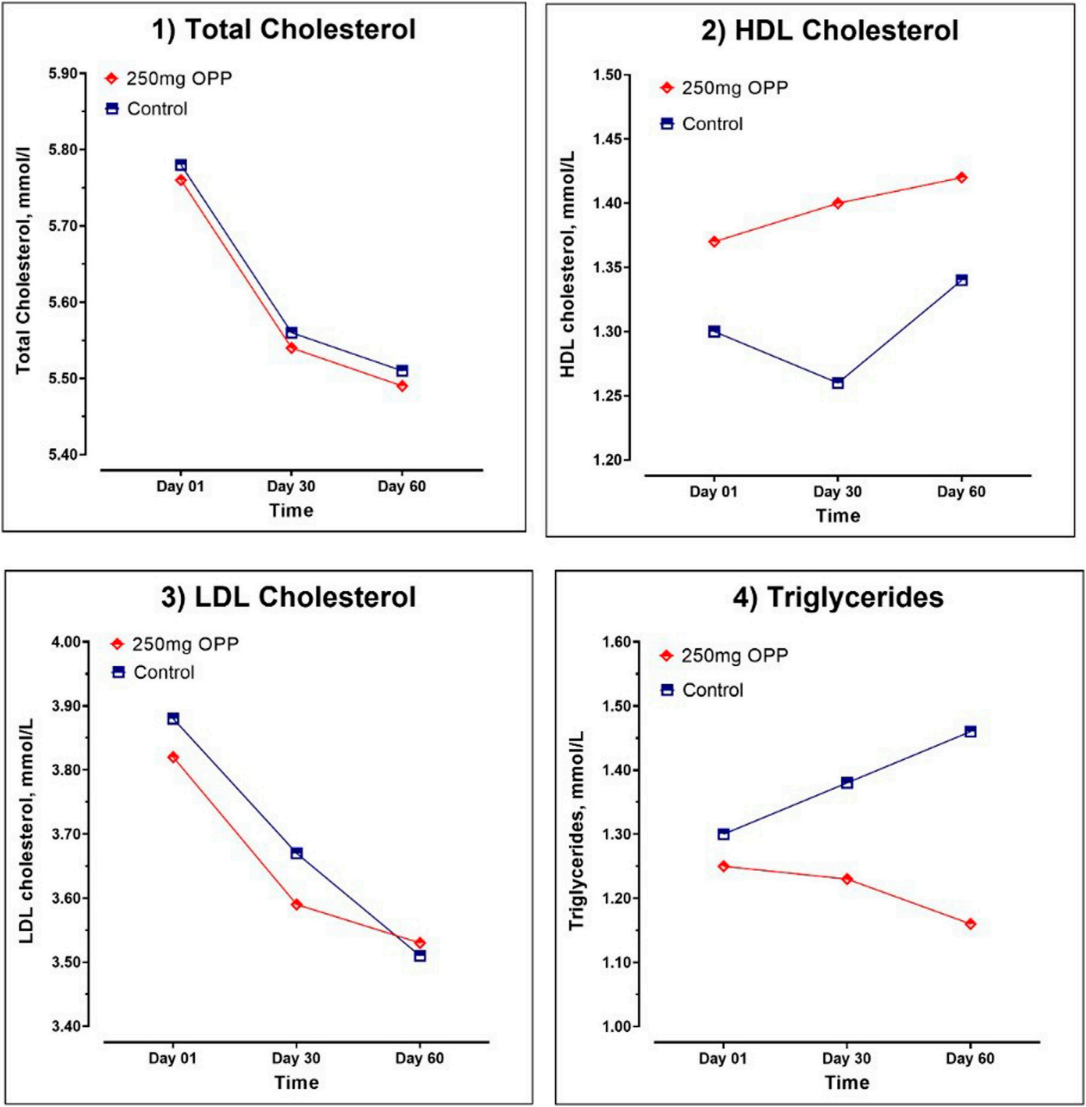
Serum lipid parameter of hyperlipidemic volunteers at baseline, day 30, and day 60 during Phase 2.

The results of the average serum lipid concentration at day 1, day 30, and day 60 are shown in Table 8; Figure 3. In the control group, serum TC (*p* = 0.018) and LDL (*p* = 0.001) significantly decreased, while TG insignificantly increased from day 1 to day 60. The HDL parameter significantly increased (*p* = 0.022) in the last 30 days of trials. As for the 250 mg OPP group, serum TC, LDL, and TG decreased, and HDL increased from day 1 to day 60. However, only TC (*p* = 0.043) and LDL (*p* = 0.031) showed significant changes. The 250 mg OPP group yielded better improvement in lipid profiles over time. However, when comparison was made using *t*-test, there was no statistically significant difference between the groups in each parameter.

**TABLE 8:** Serum lipid parameter of hyperlipidemic volunteers at baseline, day 30, and day 60 during phase 2 (mean ± SD).

	Control	250 mg OPP
Total cholesterol (mmol/L)		
Day 1	5.78 ± 0.52	5.76 ± 0.54
Day 30	5.56 ± 0.66^a^	5.54 ± 0.68^a^
Day 60	5.51 ± 0.49^a^	5.49 ± 0.67^a^
HDL (mmol/L)		
Day 1	1.30 ± 0.25	1.37 ± 0.34
Day 30	1.26 ± 0.23	1.40 ± 0.34
Day 60	1.34 ± 0.24^c^	1.42 ± 0.34
LDL (mmol/L)		
Day 1	3.88 ± 0.51	3.82 ± 0.59
Day 30	3.67 ± 0.62^a^	3.59 ± 0.63^a^
Day 60	3.51 ± 0.60^b^	3.53 ± 0.69^a^
Triglycerides (mmol/L)		
Day 1	1.30 ± 0.82	1.25 ± 0.54
Day 30	1.38 ± 0.62	1.23 ± 0.55
Day 60	1.46 ± 0.90	1.16 ± 0.46

Abbreviation: TC, total cholesterol; TG, triglycerides; LDL, low-density lipoprotein; HDL, high-density lipoprotein.

^a^Significantly different compared to day 1 within the group (*p* < 0.05).

^b^Significantly different compared to day 1 within the group (*p* < 0.001).

^c^Significantly different compared to day 30 within the group (*p* < 0.05).

Further analyses for lipid profiles was were conducted according to gender, as tabulated in Tables 9, 10 for male and female subjects, respectively. In the male subjects, although the changes in all lipid parameters in the 250 mg OPP group from baseline to day 60 showed an improvement in lipid profiles, the changes were not statistically significant. However, when the mean changes were compared between the control group and the 250 mg OPP group for each parameter, the TG level was significantly different (*p* = 0.029). This suggested that the 250 mg OPP group had more effect in improving their TG level among the male subjects.

**TABLE 9:** Mean change in lipid profile of male volunteers at baseline and 60-day follow-up (mean ± SD).

	Control (N = 11)				250 mg OPP (N = 10)				*p* ^*∼*^
	Baseline	Day 60	*p* ^*#*^	Change*	Baseline	Day 60	*p* ^*#*^	Change*	
TC (mmol/L)	5.81 ± 0.14	5.54 ± 0.17	0.133	−0.27 ± 0.17	5.83 ± 0.19	5.55 ± 0.19	0.057	−0.28 ± 0.13	0.973
HDL (mmol/L)	1.22 ± 0.05	1.28 ± 0.06	0.227	0.06 ± 0.04	1.36 ± 0.07	1.41 ± 0.09	0.106	0.05 ± 0.03	0.934
LDL (mmol/L)	4.05 ± 0.14	3.71 ± 0.16	0.049	−0.35 ± 0.15	3.90 ± 0.17	3.63 ± 0.18	0.081	−0.27 ± 0.14	0.710
Triglycerides (mmol/L)	1.16 ± 0.14	1.22 ± 0.15	0.402	0.05 ± 0.06	1.26 ± 0.17	1.01 ± 0.12	0.062	−0.25 ± 0.12	0.029

^#^Comparing mean change from baseline to 60-day follow-up.

*Change from baseline (standard error) unadjusted.

^∼^Mean change compared between groups, adjusted for baseline factor (confounder).

**TABLE 10:** Mean change in lipid profile of female volunteers at baseline and 60-day follow-up (mean ± SD).

	Control (N = 11)				250 mg OPP (N = 10)				*p* ^*∼*^
	Baseline	Day 60	*p* ^*#*^	Change*	Baseline	Day 60	*p* ^*#*^	Change*	
TC (mmol/L)	5.75 ± 0.16	5.49 ± 0.12	0.058	−0.26 ± 0.13	5.71 ± 0.13	5.45 ± 0.19	0.114	−0.26 ± 0.15	0.983
HDL (mmol/L)	1.36 ± 0.08	1.38 ± 0.07	0.497	0.02 ± 0.03	1.38 ± 0.10	1.44 ± 0.10	0.372	0.05 ± 0.06	0.628
LDL (mmol/L)	3.75 ± 0.14	3.35 ± 0.16	0.013	−0.40 ± 0.14	3.76 ± 0.16	3.46 ± 0.20	0.071	−0.30 ± 0.15	0.635
Triglycerides (mmol/L)	1.40 ± 0.27	1.65 ± 0.29	0.129	0.25 ± 0.15	1.24 ± 0.14	1.27 ± 0.13	0.857	0.03 ± 0.14	0.300

^#^Comparing mean change from baseline to 60-day follow-up.

*Change from baseline (standard error) unadjusted.

^∼^Mean change compared between groups, adjusted for baseline factor (confounder).

Another analysis for lipid profiles was according to age, as tabulated in Tables 11, 12 for adult and middle-aged subjects, respectively. In the young adults with an age range of 20–35 years old, all lipid parameters in the 250 mg OPP group showed a positive change (increased HDL and decreased TC, LDL, and TG) although the changes were not statistically significant. Meanwhile, for the middle-aged group aged between 36 and 50 years, all lipid parameters showed decreased reading from baseline to day 60 although the changes were not significant. Between the control and treated groups, there were no significant differences in both young adults and middle-aged subjects.

**TABLE 11:** Mean change in lipid profile of adult aged between 20 and 35 at baseline and 60-day follow-up (mean ± SD).

	Control (N = 11)				250 mg OPP (N = 10)				*p* ^*∼*^
	Baseline	Day 60	*p* ^*#*^	Change*	Baseline	Day 60	*p* ^*#*^	Change*	
TC (mmol/L)	5.69 ± 0.13	5.49 ± 0.13	0.066	−0.20 ± 0.10	5.77 ± 0.14	5.52 ± 0.15	0.094	−0.25 ± 0.14	0.784
HDL (mmol/L)	1.36 ± 0.07	1.42 ± 0.06	0.088	0.06 ± 0.03	1.41 ± 0.08	1.53 ± 0.08	<0.001	0.12 ± 0.03	0.148
LDL (mmol/L)	3.86 ± 0.14	3.56 ± 0.13	0.012	−0.30 ± 0.11	3.89 ± 0.12	3.59 ± 0.16	0.074	−0.30 ± 0.16	1.000
Triglycerides (mmol/L)	1.02 ± 0.12	1.11 ± 0.12	0.011	0.09 ± 0.03	1.03 ± 0.09	0.95 ± 0.08	0.458	−0.08 ± 0.10	0.143

^#^Comparing mean change from baseline to 60-day follow-up.

*Change from baseline (standard error) unadjusted.

^∼^Mean change compared between groups, adjusted for baseline factor (confounder).

**TABLE 12:** Mean change in lipid profile of elderly aged between 36 and 50 at baseline and 60-day follow-up (mean ± SD).

	Control (N = 11)				250 mg OPP (N = 10)				*p* ^*∼*^
	Baseline	Day 60	*p* ^*#*^	Change*	Baseline	Day 60	*p* ^*#*^	Change*	
TC (mmol/L)	5.93 ± 0.18	5.54 ± 0.16	0.110	−0.39 ± 0.22	5.74 ± 0.19	5.44 ± 0.26	0.105	−0.30 ± 0.17	0.746
HDL (mmol/L)	1.20 ± 0.05	1.19 ± 0.05	0.931	0.00 ± 0.04	1.32 ± 0.13	1.27 ± 0.10	0.493	−0.05 ± 0.07	0.578
LDL (mmol/L)	3.92 ± 0.16	3.41 ± 0.24	0.043	−0.51 ± 0.21	3.70 ± 0.23	3.43 ± 0.26	0.070	−0.27 ± 0.13	0.331
Triglycerides (mmol/L)	1.79 ± 0.36	2.09 ± 0.38	0.265	0.30 ± 0.25	1.58 ± 0.20	1.49 ± 0.15	0.661	−0.09 ± 0.21	0.239

^#^Comparing mean change from baseline to 60-day follow-up.

*Change from baseline (standard error) unadjusted.

^∼^Mean change compared between groups, adjusted for baseline factor (confounder).

## 4 Discussion

Our primary objective in this phase 2 clinical trial was to determine the safety of OPP supplementation on hyperlipidemic subjects by analyzing the results of routine blood tests (hematology, RFT, LFT) and by monitoring any AEs that might arise. The secondary objective was to determine the efficiencies of OPP supplementation in improving mild hyperlipidemic conditions before LLM intervention was needed. The trial was set not to be in a controlled environment since we wanted to simulate a real-life supplementation intake where the subjects could take their supplements anytime they wanted. The subjects were not allowed to take other antioxidant supplements or to participate in other trials involving intakes of supplements especially antioxidants. This was important to ensure our end results were solely due to the OPP consumption and not due to other supplements.

During the clinical trial, AEs were carefully monitored especially in the first week. There were several positive feedbacks on the subjects following the OPP consumption, among which they became more energetic (*n* = 4), their menstrual pain was reduced (*n* = 2), they could easily defecate (*n* = 7), and 4 they lose weight. No SAEs were reported that required temporary or permanent suspension of OPP. There were a few minor AEs reported, such as bloating (n = 1), increase in appetite (*n* = 4), dizziness (*n* = 2), and short menstrual duration (*n* = 2). All of the symptoms reported here were similar with our previous clinical trial (Muhammad Ismail Tadj et al., 2022). There were many *in vivo* and *in vitro* studies on the effects of OPP components, as summarized by Syarifah-Noratiqah et al. (2019). The effects on human subjects were, however, limited. We were unable to conclude if these symptoms were caused by OPP or other external causes due to the lack of literature supporting this result, besides the insignificant *t*-test results of these symptoms between the control group and the OPP group.

Moreover, the results of our blood parameters showed no abnormalities in all subjects according to the standard clinical safety references. This indicated that the OPP supplementation was safe to be orally taken by hyperlipidemic patients. In this current trial, RBC (*p* = 0.008) and PCV (*p* = 0.049) in the 250 mg OPP group significantly increased and had the same results with our phase 1 trial previously (Muhammad Ismail Tadj et al., 2022). The increases in RBC and PCV indicated that erythropoiesis (i.e., production of red blood cells) was stimulated. Literature on the effects of OPP on hematopoietic system is currently limited. However, many studies have proven that palm oil is capable in inducing erythropoiesis (Leow et al., 2011; Loganathan et al., 2017; Ermatov et al., 2019).

Other than that, RFT and LFT are the routine blood tests conducted as a part of safety assessment protocol (International Council for Harmonisation, 2016). When drugs or any substances enter a body, they will be metabolized by the hepatocytes in the liver, and then transported into the kidney and excreted as urine. Hence, if a drug consumed is harmful to the body, both of these organs will be affected earliest (Goorden et al., 2013; Rayner et al., 2016). A 50% increase of serum creatinine level from the baseline level might indicate renal failure (Kellum et al., 2021), while elevated serum ALT and AST (higher than 300 U/L and 350 U/L, respectively) indicate liver damage (Gowda et al., 2009). As for the current clinical trials, although a lot of blood parameters significantly changed over time, none of the values was above or lower the normal ranges. In fact, when *t*-test analyses between the 250 mg OPP group and the control group were conducted, none of the parameters was significantly different. Although there were a few trials on palm oil supplementation comparing between groups of healthy population and groups with specific conditions (e.g., diabetic, hyperlipidemic, obese), the blood safety parameters were rarely reported. Currently, our study was the only human trial conducted to determine the blood safety parameters of palm oil supplementation on the hyperlipidemic population. The previous clinical trial of palm oil supplementation on healthy population (Fairus et al., 2018; Lv et al., 2018; Muhammad Ismail Tadj et al., 2022) and diabetic population (Tan et al., 2018) for 2 months also showed no abnormalities in the hematology, renal, and liver profiles.

Hyperlipidemia is described by TC, LDL, or TG levels greater than the 90th percentile in comparison to the general population, or an HDL level less than the 10th percentile compared to the general population (Fredrickson, 1971). In this present clinical trial, the efficiency of OPP in improving hyperlipidemic condition was measured by increased serum HDL and reduced serum TC, LDL, and TG compared to the control group. Our findings showed none of the lipid parameters in the 250 mg OPP group was significantly different from the control group. The 3-month palm oil supplementation human clinical trial by Lucci et al. (2016) also showed the same finding where TC, LDL, and HDL between control group and 25 mL/day of hybrid palm oil-rich diet was statistically insignificant.

While the declining patterns of TC and LDL in both groups were very similar, only the TG parameter showed the opposite pattern. Thus, we decided to make another analysis to determine if the OPP supplementation affected the serum TG levels in other conditions. Since lipid circulating fraction is strongly affected by individual characteristics such as age and gender (Russo et al., 2015), we decided to further analyze our lipid results based on these two factors. We found that the serum TG levels in the male subjects from the 250 mg OPP group significantly decreased (mean changes, −0.25 ± 0.12; *p*-value, 0.029) compared to the male subjects in the control group. Several studies have claimed that palm oil supplementation in male subjects were capable in improving serum TG levels among induced pathological conditions such as hyperlipemia or diabetes (Rosalina Tan et al., 2011; Olabiyi et al., 2021). The other analyses of lipid parameters on female, adult, and elderly subjects did not reveal any statistically significant results. There was a possibility that the OPP supplementation might be able to improve the serum TG levels in the hyperlipidemic population, especially among males.

The efficacy results from the randomized controlled trials can be heavily affected by sharing early treatment-response results on ongoing clinical trials (Ventz et al., 2021), which was practiced in this trial. There were many disadvantages if the results were to be released early, such as low enrollment rate (Fleming et al., 2008) that might lead to trial termination (Green et al., 1987), increased drop-out rate especially when the interim results did not satisfy the subjects’ expectation (White et al., 2011), which then affected the clinicians’ recommendations and thus subjects’ decision-making (Berry, 2015). All these events might lead to altered subjects’ demographics and clinical profiles and increased probability of compromising the statistical validity (Ventz et al., 2021). We released the health examination results (which consisted of hematology, renal, liver, and lipid profile test results) after the intervention days to the subjects. This action might have influenced the subjects’ dietary intake, thus affecting their lipid profiles.

There was a possibility that if the subjects noticed that their lipid profiles were abnormally high, they would regulate their dietary intake so that the level would return to normal. The lipid parameters can be easily altered by healthy diets, especially with the help from nutritionists and food guidelines such as Dietary Approaches to Stop Hypertension (DASH) eating plan (Patton, 2013; Campbell, 2017). This might explain the similar pattern of lipid profiles between the control group and the 250 mg OPP group. While withholding interim data can solve this problem, this practice is ethically wrong (Collier, 2015) since the subjects had the right to know the results. Hence, what we can do for future research to ensure the efficacy of treatments and not to be affected by the subjects’ decision-making is by developing a result notification guideline.

A recommended method by Fernandez et al. (2012) is to include the mechanism by which the subjects will be offered results, the anticipated timing of the release of results, the potential risks (emotional or any other forms of harms) and benefits of receiving results, and plans to support the subjects. For example, in this trial, all the subjects received their written medical reports a few days after the intervention day. Hence, it was not compulsory for them to meet the physicians and thus no important discussions with regard to future plans were exchanged. According to the recommendation, medical reports received by each subject should be discussed with the physicians in-charge. In addition, the trial staff or the physicians should state that dietary and lifestyle patterns should remain unchanged throughout the entire trial to ensure any changes in the medical reports should only be affected by the supplement intake.

Another limitation in our trial is the enrollment of small sample size; thus, the research was not powered enough to deliver definitive conclusions on the end points related to measured parameters. One of the disadvantages of clinical studies with small sample size is, the outcomes that are statistically significant may not be as generalizable because the situation in which the rules apply may be narrower than those for bigger clinical studies with identical probabilities (*p* values) (Evans and Ildstad, 2001). An appropriate designed small clinical study, however can contribute to valuable evidence of efficacy; although the conclusions made may require the use of assumptions and inferences given the paucity of data generated (Siegel, 2000). Future studies should evaluate the efficacy of OPP in improving hyperlipidemia condition in larger and more diverse population (multi-center clinical trials).

## 5 Conclusion

Consumption of 250 mg OPP daily by hyperlipidemic subjects is safe and significantly lowers the serum triglycerides among male hyperlipidemic subjects.

## Data Availability

The raw data supporting the conclusion of this article will be made available by the authors, without undue reservation.
